# Supplementary material for: A distributed fMRI-based signature for the subjective experience of fear
Source: Nat Commun. 2021 Nov 17;12:6643. doi: 10.1038/s41467-021-26977-3 (PMC8599690; doi:10.1038/s41467-021-26977-3)
Supplement: Supplementary file 1 — Editor Summary [file 41467_2021_26977_MOESM1_ESM.docx]

The brain systems underlying fear experience are debated. Here the authors develop an fMRI-based neural signature for fear and show that fear is represented in distributed brain systems rather than isolated ‘fear centers’.
