# Supplementary material for: A distributed fMRI-based signature for the subjective experience of fear
Source: Nat Commun. 2021 Nov 17;12:6643. doi: 10.1038/s41467-021-26977-3 (PMC8599690; doi:10.1038/s41467-021-26977-3)
Supplement: Supplementary file 4 — Reporting Summary [file 41467_2021_26977_MOESM4_ESM.pdf]

nature research

Corresponding author(s): Benjamin Becker and Tor D. Wager

Last updated by author(s): Oct 12, 2021

Reporting Summary

Nature Research wishes to improve the reproducibility of the work that we publish. This form provides structure for consistency and transparency in reporting. For further information on Nature Research policies, see our Editorial Policies and the Editorial Policy Checklist.

Statistics

For all statistical analyses, confirm that the following items are present in the figure legend, table legend, main text, or Methods section.

n/a	Confirmed	
☐	☒	The exact sample size (n) for each experimental group/condition, given as a discrete number and unit of measurement
☐	☒	A statement on whether measurements were taken from distinct samples or whether the same sample was measured repeatedly
☐	☒	The statistical test(s) used AND whether they are one- or two-sided<br>*Only common tests should be described solely by name; describe more complex techniques in the Methods section.*
☐	☒	A description of all covariates tested
☐	☒	A description of any assumptions or corrections, such as tests of normality and adjustment for multiple comparisons
☐	☒	A full description of the statistical parameters including central tendency (e.g. means) or other basic estimates (e.g. regression coefficient) AND variation (e.g. standard deviation) or associated estimates of uncertainty (e.g. confidence intervals)
☐	☒	For null hypothesis testing, the test statistic (e.g. F, t, r) with confidence intervals, effect sizes, degrees of freedom and P value noted<br>*Give P values as exact values whenever suitable.*
☒	☐	For Bayesian analysis, information on the choice of priors and Markov chain Monte Carlo settings
☐	☒	For hierarchical and complex designs, identification of the appropriate level for tests and full reporting of outcomes
☐	☒	Estimates of effect sizes (e.g. Cohen's d, Pearson's r), indicating how they were calculated

Our web collection on statistics for biologists contains articles on many of the points above.

Software and code

Policy information about availability of computer code

Data collection	Stimuli were presented using the E-Prime software (Version 2.0; Psychology Software Tools, Sharpsburg, PA), MRI data were acquired using validated standard protocols on a GE MRI system
Data analysis	Preprocessing, first level and second level modelings of fMRI data were performed using the SPM toolbox (SPM12 v7487, https://www.fil.ion.ucl.ac.uk/spm/software/spm12/). Multivariate pattern analysis was conducted using the CANLab Core tools (https://github.com/canlab/CanlabCore; version 2-18-2020). Both SPM12 and CANLab Core tools were implemented in MATLAB 2015b.

For manuscripts utilizing custom algorithms or software that are central to the research but not yet described in published literature, software must be made available to editors and reviewers. We strongly encourage code deposition in a community repository (e.g. GitHub). See the Nature Research guidelines for submitting code & software for further information.

Data

Policy information about availability of data

All manuscripts must include a data availability statement. This statement should provide the following information, where applicable:

- Accession codes, unique identifiers, or web links for publicly available datasets
- A list of figures that have associated raw data
- A description of any restrictions on data availability

The key data is available on https://figshare.com/articles/dataset/Subjective_fear_dataset/13271102, other data is available upon request from the authors

Field-specific reporting

Please select the one below that is the best fit for your research. If you are not sure, read the appropriate sections before making your selection.

☒ Life sciences ☐ Behavioural & social sciences ☐ Ecological, evolutionary & environmental sciences

For a reference copy of the document with all sections, see nature.com/documents/nr-reporting-summary-flat.pdf

Life sciences study design

All studies must disclose on these points even when the disclosure is negative.

Sample size	No statistical tests were used to predetermine the sample size, but this sample size is within the standard range and in accordance with the suggestions in the field (see e.g., Kohoutová et al., 2020). To further validate the robustness of the findings independent replication and generalization datasets were included.
Data exclusions	3 participants were excluded due to the excessive head motion (> 1 voxel) during fMRI scanning data.
Replication	Cross-validation (and testing in another two independent samples) showed that the effects replicated across individuals and studies.
Randomization	Not applicable. The current study only included one group thus no randomization was performed.
Blinding	Given that no between subject design was employed data collection and analysis were not performed blind to the conditions of the experiments.

Reporting for specific materials, systems and methods

We require information from authors about some types of materials, experimental systems and methods used in many studies. Here, indicate whether each material, system or method listed is relevant to your study. If you are not sure if a list item applies to your research, read the appropriate section before selecting a response.

Materials & experimental systems

n/a	Involved in the study	
☒	☐	Antibodies
☒	☐	Eukaryotic cell lines
☒	☐	Palaeontology and archaeology
☒	☐	Animals and other organisms
☐	☒	Human research participants
☒	☐	Clinical data
☒	☐	Dual use research of concern

Methods

n/a	Involved in the study	
☒	☐	ChIP-seq
☒	☐	Flow cytometry
☐	☒	MRI-based neuroimaging

Human research participants

Policy information about studies involving human research participants

Population characteristics	Discovery cohort: N=67 (34 females; mean ± SD age = 21.5 ± 2.1 years); Validation cohort: N=20 (6 females; mean ± SD age = 21.75 ± 2.61 years); Generalization cohort: N=31 (15 females; mean ± SD age = 23.29 ± 4.21 years).
Recruitment	Healthy volunteers were recruited by means of local advertisement. Exclusion criteria included color blindness; current or regular substance or medication use; current or history of medical or psychiatric disorders; any contraindications for MRI. We do not expect self-selection biases to substantially influence findings.
Ethics oversight	All participants in the discovery and validation cohorts provided written informed consent, and the study was approved by the local ethics committee at the University of Electronic Science and Technology of China and was in accordance with the most recent revision of the Declaration of Helsinki. Participants in the generalization cohort provided written informed consent and the study was approved by the Institutional Review Board of Advanced Telecommunications Research Institute International (ATR), Japan.

Note that full information on the approval of the study protocol must also be provided in the manuscript.

Magnetic resonance imaging

Experimental design

Design type	Task-based fMRI
Design specifications	Discovery cohort: 80 trials evenly distributed in 4 runs, 6 second stimulus, 4 second rating; validation cohort: 60 trials evenly distributed in 2 runs, 6 second stimulus, 4 second rating; generalization cohort: 3600 trials, 1 second stimulus.
Behavioral performance measures	Subjective fear rating.

Acquisition

Imaging type(s)	functional
Field strength	3.0 Tesla (all cohorts)
Sequence & imaging parameters	Functional MRI data for the discovery and validation paradigms was acquired using a T2*-weighted echo-planar imaging (EPI) pulse sequence (repetition time = 2s, echo time = 30ms, 36 slices, slice thickness = 3.8mm, no gap, field of view = 200 × 200mm, resolution = 64 × 64, flip angle = 90°, voxel size = 3.125 × 3.125 × 3.8mm). Generalization cohort: 33 contiguous slices (Repetition time (TR) = 2000 ms, Echo time (TE) = 30 ms, voxel size = 3 × 3 × 3.5mm3, field-of-view = 192 × 192 mm, matrix size = 64 × 64, slice thickness = 3.5 mm, 0 mm slice gap, flip angle = 80°) oriented parallel to the AC-PC plane.
Area of acquisition	Whole brain

Diffusion MRI ☐ Used ☒ Not used

Preprocessing

Preprocessing software	SPM12 (v7487)
Normalization	Nonlinear deformation based on anatomical data.
Normalization template	ICBM152 space
Noise and artifact removal	First level models included motion parameter estimates.
Volume censoring	For discovery and validation cohorts, outlier timepoints were included in the first level models as nuisance regressors.

Statistical modeling & inference

Model type and settings	First level models were mass-univariate, second level analyses included both univariate and multivariate models.
Effect(s) tested	Linear parametric effect of fear ratings; Prediction of subjective fear rating.

Specify type of analysis: ☒ Whole brain ☐ ROI-based ☐ Both

Statistic type for inference (See Eklund et al. 2016)	voxel-wise
Correction	FDR

Models & analysis

n/a	Involved in the study
☒	☐ Functional and/or effective connectivity
☒	☐ Graph analysis
☐	☒ Multivariate modeling or predictive analysis

Multivariate modeling and predictive analysis	Support vector regression model predicting subjective fear rating. Features included activation to each fear rating. Prediction-outcome correlation and Cohen's d were estimated using cross-validation and independents samples.
